# Synthesis and Characterization of a Binuclear Copper(II)-dipyriamethyrin Complex: [Cu_2_(dipyriamethyrin)(μ_2_-1,1-acetato)_2_]

**DOI:** 10.3390/molecules25061446

**Published:** 2020-03-23

**Authors:** James T. Brewster, Harrison D. Root, Hadiqa Zafar, Gregory D. Thiabaud, Adam C. Sedgwick, Jiaming He, Vincent M. Lynch, Jonathan L. Sessler

**Affiliations:** 1 Department of Chemistry, The University of Texas at Austin 105 E 24th Street-Stop A5300, Austin, TX 78712-1224, USAhroot@utexas.edu (H.D.R.); hzafar@mit.edu (H.Z.); gtamanaco@msn.com (G.D.T.); a.c.sedgwick@utexas.edu (A.C.S.); vmlynch@cm.utexas.edu (V.M.L.); 2 Materials Science and Engineering Program and Texas Materials Institute, The University of Texas at Austin, Austin, TX 78712, USA; jiaminghe@utexas.edu

**Keywords:** dipyriamethyrin, expanded porphyrin, binuclear copper, porphyrinoid, allosteric effect

## Abstract

The reaction between dipyriamethyrin and copper(II) acetate [Cu(OAc)_2_] afforded what is, to our knowledge, the first transition metal-dipyriamethyrin complex. Molecular and electronic characterization of this binuclear Cu(II) complex via EPR, UV-vis, and single crystal X-ray diffraction analysis revealed marked differences between the present constructs and previously reported binuclear copper(II) hexaphyrin species. UV-vis titration analyses provided evidence for a homotropic positive allosteric effect, wherein the binuclear species is formed without significant intermediacy of a monomeric complex.

## 1. Introduction

The incorporation of specific metal ions within enzyme active sites facilitates numerous chemical transformations required for proper biological function [1,2,3]. Mutation of the amino acids defining the cation coordination sphere can lead to structural modification of the core metal complex geometry with concomitant loss or gain of function [4,5]. Non-native enzymatic systems, prepared by switching important amino acid residues or the coordinated metal ion, have provided mechanistic insights into the action of many metalloenzymes [6]. Concurrent with these efforts, non-natural and synthetic model systems have allowed for the controlled modulation of desired properties and provided access to new chemistry [7,8,9,10,11].

**Figure 1:**
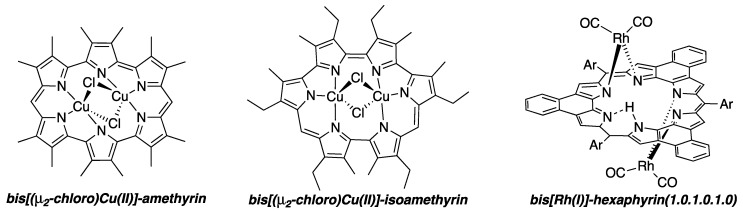
Representative binuclear hexaphyrin complexes.

Tetrapyrrolic porphyrins and related congeners have found widespread utility as mononuclear metalloenzyme active site mimics with attendant applications in catalysis, among other uses [12,13,14]. Extension of these efforts to multiple metal and main group ion complexes remains rare. Indeed, only a few examples of binuclear complexes, such as Rh, Hg, Re, Tc, B, and alkali metal ions, have been structurally characterized [15,16,17,18,19,20,21,22]. Expanded porphyrinoids, frameworks containing a larger internal cavity, have extended the chemistry of such systems via the stabilization of multiple metal ions coordinated within a single lacuna [23,24]. Within this paradigm, expanded and related non-conjugated larger porphyrinoid systems have yielded a diverse array of homo- and hetero-binuclear transition metal and main group complexes [22,25,26,27,28,29,30,31,32,33,34,35,36,37,38,39,40,41,42,43,44,45]. Previous efforts by our group using hexaphyrin expanded porphyrins have also resulted in the synthesis and characterization of binuclear copper complexes (Figure 1) [18,46,47,48]. In these instances, modification of the heterocyclic components within the hexaphyrin core has allowed for subtle changes in the electronic structure and metal-metal communication.

Recent efforts with the so-called dipyriamethyrin ligand (**1**), a bis-pyridine hexaaza-porphyrinoid, yielded a series of thorium(IV), uranium(IV), neptunium(IV), and uranyl(VI) complexes [49,50]. However, transition metal complexes have yet to be reported using this hexaphyrin analogue. We envisaged that the mixed heterocyclic coordinating motifs present in dipyriamethyrin **1** might confer new properties to a transition metal complex distinct from previous hexaphyrin species. Here, we report the synthesis and characterization of a binuclear [Cu_2_(dipyriamethyrin)(μ_2_-1,1-acetato)_2_] complex (**2**). The molecular and electronic properties of **2** were characterized by UV-vis and EPR spectroscopy, cyclic voltammetry, and single crystal X-ray diffraction analysis. 

## 2. Results and Discussion

**Scheme 1:**
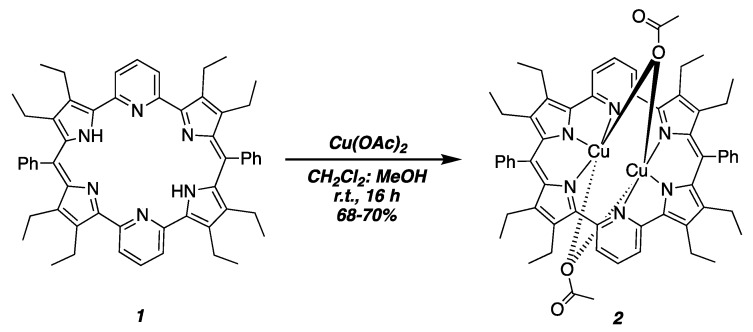
Synthesis of binuclear copper(II) complex **2**.

The dipyriamethyrin of this study was prepared using our recently optimized procedure [50]. Treatment of the free-base form of **1** with copper acetate [Cu(OAc)_2_] (25 equiv.) in a methanol: dichloromethane mixture (1:1, *v*/*v*) open to the laboratory atmosphere at room temperature for 16 h yielded the dinuclear copper complex **2** as a purple solid with green metallic lustre in 68–70% yield, after purification by filtration over Celite and crystallization from dichloromethane: hexanes (Scheme 1 & ESI Figure S1). The purified complex **2** proved unstable under prolonged exposure of protic and non-anhydrous solvents, reverting back to the free-base ligand (**1**) or a mixture of complex **2** and ligand **1**, respectively. Complex **2** also proved unstable under conditions of silica gel, neutral aluminum oxide, and basic aluminium oxide purification. However, **2** proved sufficiently stable in neutral, apolar media to allow for characterization, including via single crystal X-ray diffraction analysis. Interestingly, the equilibrium between metallation and protonation observed with complex **2** was not seen with related amethyrin and (naphtho)isoamethyrin species [46,47,48]. In the case of previous hexaphyrin ligands, formation of the binuclear Cu(II) complex was believed to occur with a concomitant 2π-electron oxidation yielding a globally aromatic porphyrinoid ligand. No such redox chemistry was observed in the case of dipyriamethyrin and may contribute to the metal complex lability.

**Figure 2:**
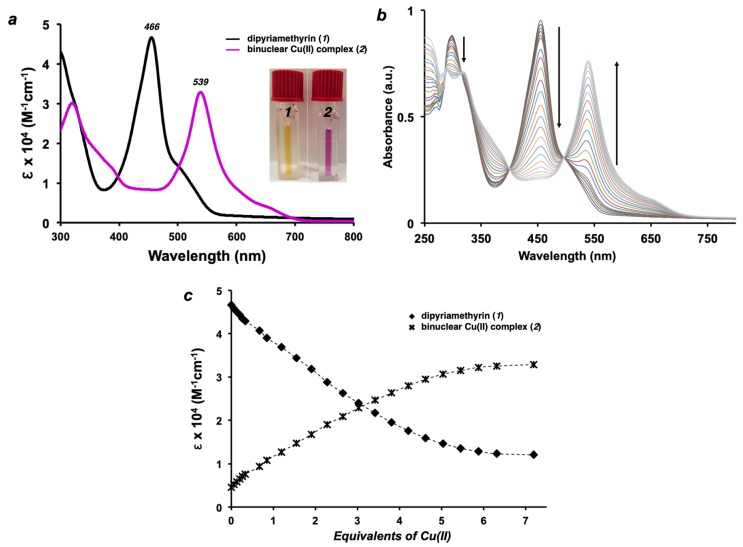
(**a**) UV-vis spectrum of ligand **1** (black) and complex **2** (pink) in THF. The insert shows photographs of **1** and **2** as solutions in tetrahydrofuran. (**b**) UV-vis titration data corresponding to the conversion of the free-base dipyriamethyrin **1** (9.4 μM) to the corresponding binuclear Cu(II) complex **2** upon the addition of Cu(II)(OAc)_2_ in THF and (**c**) changes in the molar absorptivity (ε) at the maximum wavelength (λ_max_ of **1** = 466 nm; λ_max_ of **2** = 539 nm) plotted against equivalents of Cu(II)(OAc)_2_.

Upon metalation, an easy-to-visualize solution-state colour change from yellow-orange to pink-purple was observed (Figure 2a). The UV-vis spectral profile, as measured in tetrahydrofuran, displayed a slight decrease in the molar absorptivity from ε = 46600 M^−1^ cm^−1^ to ε = 32900 M^−1^ cm^−1^ with a bathochromic shift in the Soret-band (*λ*_max_ = 539 nm). Dipyriamethyrin (**1**) is not fully through conjugated, i.e., globally aromatic. In terms of its electronic features, it thus behaves more similar to dipyrromethene with the major transition (*λ*_max_ = 466) being assigned as a charge transfer process associated with the π-system of the dipyrrin subunit [51,52,53]. The large absorption in dipyriamethyrin (**1**) and the binuclear copper complex (**2**) are attributed to the dipyriamethyrin π-π* transition [54]. Previous studies on transition metal complexes also report a bathochromic shift to the *λ*_max_, suggesting a metal-mediated reduction in the HOMO-LUMO gap [52,53,54,55]. Furthermore, in analogy to studies on Th(IV), U(IV), and Np(IV)-dipyriamethyrin complexes, the broadening of the Soret-like band in complex **2** may be due to an overlapping metal-to-ligand charge transfer (MLCT) transition [50]. UV-vis titration analysis using dipyriamethyrin (9.4 μM) in THF and Cu(OAc)_2_ (0.33 mM stock solution in THF) showed direct conversion from the free-base **1** to the binuclear Cu(II) complex **2** (Figure 2b,c). Formation of **2** is posited to arise via the intermediacy of a sitting atop (SAT) complex followed by a homotropic positive allosteric process wherein the first cation complexation event serves to activate the dipyriamethyrin ring for the second binding event [20,55,56,57,58,59,60]. Evidence for a potential positive allosteric (i.e., acetate directing) effect was also gleaned from X-ray crystallographic data (ESI Figure S2). Formation of the initial mono-metallated complex is postulated to arise through cooperation between the Cu(II) cation and acetate counter by a concerted metalation-deprotonation (CMD)-type mechanism (cf. ESI, Scheme 1) [61,62,63].

**Figure 3:**
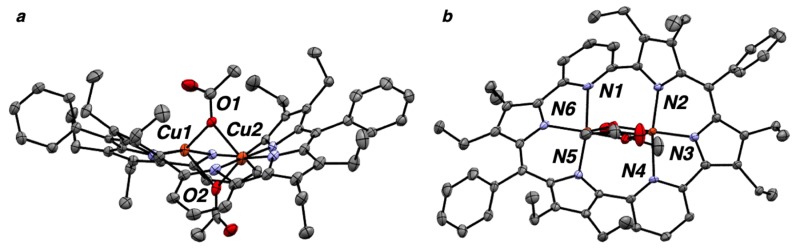
Single crystal X-ray structures of complex **2** viewed from (**a**) side and (**b**) top perspective. Hydrogen atoms are omitted for clarity.

Crystals suitable for X-ray diffraction analysis were grown via evaporation of a dichloromethane: hexanes (1:2, *v*/*v*) solution of **2**. As illustrated in Figure 3, the binuclear copper complex (**2**) adopts a twisted conformation so as to complex the two Cu(II) ions effectively. Torsion within the porphyrinoid ring arises from a twist between the biaryl (*C*_sp_^2^-*C*_sp_^2^) pyridine-pyrrole heterocyclic subunits (i.e., N1 to N2 and N4 to N5). In analogy to previous binuclear Cu(II)-amethyrin complexes, all six heterocycles participate in coordination to the complexed metal ions. The coordination environment of Cu1 is defined by an equatorial pyrrol-2-ylidene (N6) and 1,1-acetates (O1 and O2) with additional axial pyridine (N1) and pyrrole (N5) donors. A similar first coordination sphere is found for Cu2 with N3, O1, and O2 defining the equatorial plane and N2 and N4 defining the axial plane. 

Following the approach of Addison and co-workers, wherein the structural parameter τ_5_ is calculated as an index of trigonality with τ_5_ = 1 being defined as an ideal trigonal bipyramidal geometry [64], the bis-copper(II) complex **2** is characterized by τ_5_ = 0.69 for Cu1 and 0.73 for Cu2, respectively [65]. These values represent a lower level of distortion than related amethyrin species, a finding attributed to the enhanced flexibility of the dipyriamethyrin core [50]. The nitrogen-Cu(II) distances reveal an asymmetric coordination environment and are 2.107(2) Å (N1-Cu1), 1.987(3) Å (N2-Cu2), 1.889(2) Å (N3-Cu2), 2.080(2) Å (N4-Cu2), 1.998(2) Å (N5-Cu1), and 1.867(2) Å (N6-Cu1), respectively. The Cu(II)-Cu(II) distance, 3.0276(6) Å, is longer than that found in the case of the corresponding complexes of amethyrin (2.761(1) Å), isoamethyrin (2.744(2) Å), and naphthoisoamethyrin (2.752 Å). Unlike the binuclear Cu(II) complex of amethyrin, the core structure of **2** contains bridging acetates. The four Cu-O bond distances are nonequivalent at 1.971(4) Å (O1-Cu1) and 2.181(4) Å (O1-Cu2) and 2.134(2) Å (O2-Cu1) and 1.968(2) Å (O2-Cu2). The bond angles (θ) of the μ_2_-1,1-acetates are 93.36° for Cu1-O1-Cu2 and 94.90° for Cu1-O2-Cu2, respectively. Single crystal X-ray crystallographic analysis of the binuclear copper(II)-dipyriamethyrin system also revealed a mixture of μ2-acetate and μ2-chloride anions bound to the Cu(II) ions. The site occupancy factor for the chloride ion refined to 11%. Similar acetate-chloride ligand exchange has been seen with the related isoamethyrin and naphthoisoamethyrin binuclear copper(II) complexes [47,48] Attempts to prevent ligand exchange by using different solvent systems (i.e., THF, THF: hexanes, toluene, and toluene: hexanes) failed to yield X-ray diffraction grade single crystals. Attempts to convert **2** from the partial bis-bridging acetate complex to the fully bis-bridging chloride species (i.e., by prolonged solvation in CH_2_Cl_2_ exposed to light) also failed to yield X-ray quality single crystals. 

**Figure 4:**
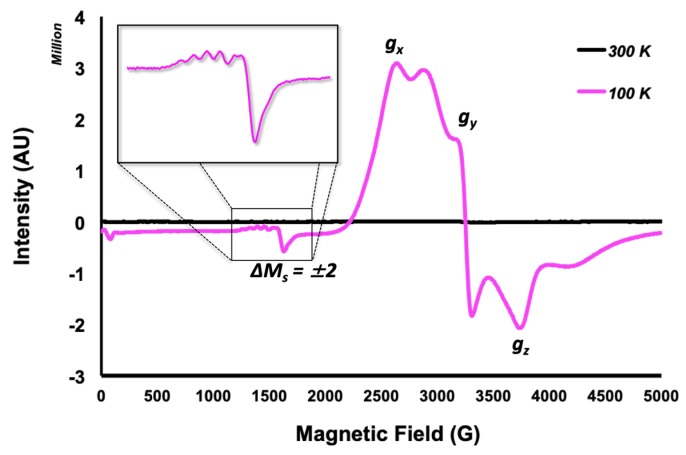
Variable temperature X-band EPR spectra at 300 K (black) and 100 K (purple) of complex 2 recorded in toluene (in solution and frozen glass, respectively).

EPR spectroscopic studies using freshly prepared complex **2**, carried out at 100 K in frozen toluene, revealed features readily assignable to two coupled copper(II) ions [66]. The spectrum of **2** yielded an EPR pattern for the triplet state (*S* = 1) with both Δ*M*_s_ ± 1 and Δ*M*_s_ ± 2 (half-field transition; *g* = 4.15) features being observed (Figure 4). This is consistent with a pair of copper(II) ions. To probe the putative electronic exchange between the copper ions, analogous studies were carried out at 300 K in toluene solution. In this case, the signal intensity decreased significantly and was devoid of discernible signals. Presumably, this reflects a lower level of coupling under these latter higher temperature conditions. Similar temperature dependence was seen with amethyrin and naphthoisoamethyrin, as well as simpler fused and monomeric porphyrin constructs [46,48,67,68].

In conclusion, we have prepared a binuclear copper(II) dipyriamethyrin complex displaying a unique molecular and electronic structure that differs from bimetallic complexes formed from other hexaphyrin-type expanded porphyrins. These differences are ascribed to the presence of the incorporated pyridine motifs, lack of global aromaticity between the dipyrromethene and pyridine components, and the flexible cavity provided by dipyriamethyrin. The present work thus serves to highlight how changes in the coordinating motifs within a macrocyclic ligand framework can alter the structural features and chemical properties of multinuclear metal complexes. The information gleaned from this work further advances our design understanding of what is needed to support a specific binuclear transition metal ion-expanded porphyrinoid complexation motifs. It thus sets the stage for further work in what is likely to be a rich subfield lying at the nexus of expanded porphyrin and inorganic coordination chemistry. 

## 3. Materials and Methods 

All reagents and solvents were purchased from Sigma Aldrich (Milwaukee, WI, USA) and ACROS Organics (Morris Plains, NJ, USA) and used without further purification unless otherwise noted. Column chromatography was carried out using basic alumina (aluminum oxide, basic, Brockmann 1, 50–200 μm, 60 A; ACROS Organics (Morris Plains, NJ, USA)). High-resolution mass spectrometric (HRMS) measurements were conducted by Dr. Ian Riddington and co-workers in The University of Texas at Austin Department of Chemistry Mass Spectrometry Facility using an Ion Spec Fourier Transform mass spectrometer (Agilent Technologies 6530 Accurate-Mass Q-TOF LC/MS, 9.4 T, USA). UV-Vis spectra were recorded from 250 to 800 nm using a Varian Cary 5000 spectrophotometer (Agilent, USA) at room temperature. A cell length of 10 mm was used for all UV-Vis spectral studies. Variable temperature (VT) electron paramagnetic resonance (EPR) spectroscopy at X-band (9.5 GHz) was carried out using a Bruker EMX Plus spectrometer (USA). Experimental settings consisted of an amplitude modulation = 10 G and a microwave power = 2 mW. The time constant was set to 20.48 msec with a 40.00 msec conversion time and 160.00 sec sweep time. The resolution in X was set to 4000 and final spectrum were an average of 8 scans. Saturated solutions of **2** in anhydrous toluene were used for the EPR analyses and studied either as a solution or frozen glass as dictated by the choice of measurement temperature. 

*Synthesis of Binuclear Copper Complex* (**2**). To a scintillation vial charged with a 5 mm PTFE stir bar and dipyriamethyrin (**1**) [26] (50 mg, 0.061 mmol) was added Cu(OAc)_2_ (279 mg, 1.53 mmol, 25 equiv.) and 10 mL of a methanol: CH_2_Cl_2_ (1:1, *v*/*v*) mixture. The reaction vessel was capped and stirred at room temperature for 16 h. After this time, the solvent was removed under a stream of nitrogen. The solid obtained in this way was suspended in 5 mL of dichloromethane and filtered through a glass pipette plug of Celite into a clean scintillation vial. The reaction vial was washed first with 2.5 mL of CH_2_Cl_2_ and again with 5 mL of a dichloromethane: hexanes (1:4, *v*/*v*) mixture, which were also filtered through the Celite plug. The organics were combined into a 20 mL scintillation vial, diluted with an additional 5 mL of hexanes, and allowed to sit loosely capped in the dark overnight. The resultant material was filtered to yield **2** as a dark purple solid with green metallic luster (44–45 mg, 68–70%). Single crystals suitable for X-ray diffraction analysis were grown by dissolving ca. 10 mg of **2** into 2.5 mL of a CH_2_Cl_2_: hexanes (1:2, *v*/*v*) mixture. As noted in the main body of the text, attempts to purify complex **2** *via* silica gel, neutral aluminum oxide, or basic aluminum oxide column chromatography resulted in loss of product on the column or reversion to the free base ligand (**1**).

## Supplementary Materials

The following are available online, Figure S1: Photograph of binuclear copper(II) complex **2** (CCDC No. 1984261). Figure S2: Single crystal X-ray diffraction data showing additional coordination of the axial acetate carbonyl oxygen to Cu(II). Scheme S1: Proposed positive allosteric mechanism for the formation of binuclear copper(II) complex **2**. Figure S3: .cif and X-ray crystallographic experimental data. Table S1. Crystal data and structure refinement for **2**. Table S2. Atomic coordinates and equivalent isotropic displacement parameters for **2**. Table S3. Bond lengths and angles for **2**. Table S4. Anisotropic displacement parameters for **2**. Table S5. Hydrogen coordinates and isotropic displacement parameters for **2**. Table S6. Torsion angles for **2**.
