# Supplementary material for: Synthesis and Characterization of a Binuclear Copper(II)-dipyriamethyrin Complex: [Cu2(dipyriamethyrin)(μ2-1,1-acetato)2]
Source: Molecules. 2020 Mar 23;25(6):1446. doi: 10.3390/molecules25061446 (PMC7145289; doi:10.3390/molecules25061446)

Electronic Supplementary Information

Synthesis and characterization of a binuclear copper-dipyriamethyrin complex: [Cu_2(dipyriamethyrin)(μ_2-1,1-acetato)$_2$]

James T. Brewster II,[a] Harrison D. Root,[a] Hadiqa Zafar,[a] Gregory D. Thiabaud,[a] Adam C. Sedgwick,[a] Jiaming He,[b] Vincent M. Lynch,[a] and Jonathan L. Sessler*,[a]

[a]Department of Chemistry, The University of Texas at Austin, Texas 78712-1224, USA
[b]Materials Science and Engineering Program and Texas Materials Institute, The University of Texast at Austin, Austin, Texas 78712, USA
sessler@cm.utexas.edu

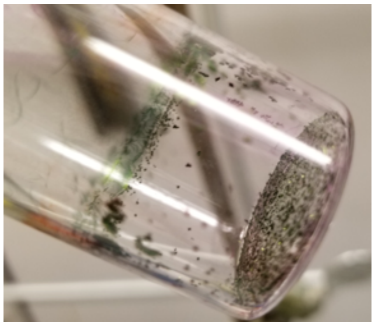

Figure S1. Photograph of residual single crystals of binuclear copper(II) complex **2** (CCDC No. 1984261).

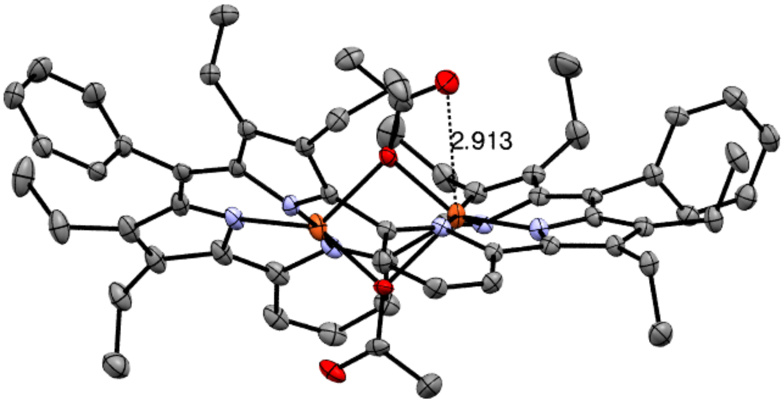

Figure S2. Single crystal X-ray diffraction data showing a long, possible bond of the axial acetate carbonyl oxygen to Cu(II). Displacement ellipsoids are scaled to the 50% probability level. Hydrogen atoms are omitted for clarity.

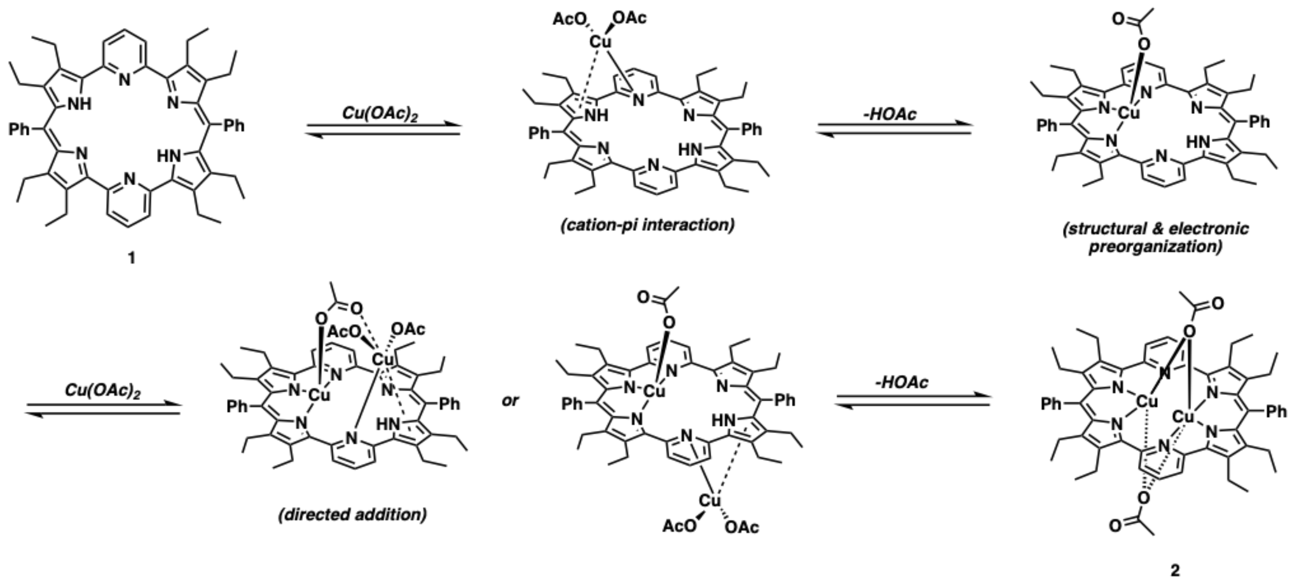

Scheme S1. Proposed positive allosteric mechanism for the formation of binuclear copper(II) complex **2**.

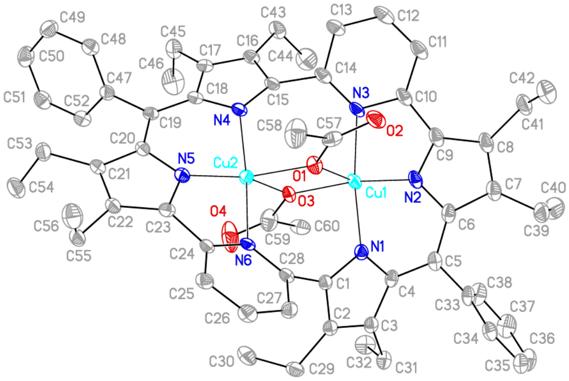

Figure S3. View of binuclear Cu(II) complex in **2** showing the atom labeling scheme. Displacement ellipsoids are scaled to the 50% probability level. The hydrogen atoms and the lower occupancy atoms of the disordered groups were omitted for clarity.

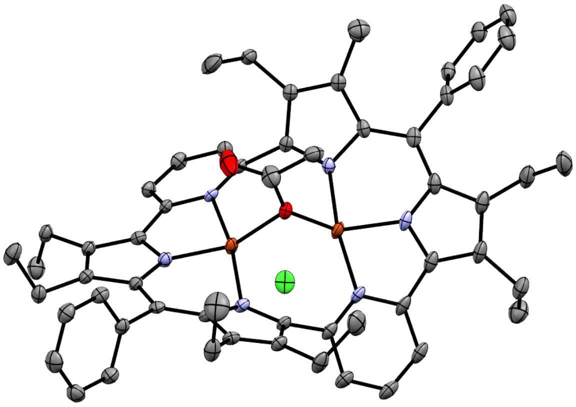

Figure S4. Single crystal X-ray diffraction data showing the mixed chloro-acetato binuclear Cu(II) complex. Displacement ellipsoids are scaled to the 50% probability level. Hydrogen atoms were omitted for clarity.

X-ray Experimental for **2**: Crystals grew as thin, green laths by slow evaporation from CH_2Cl_2: hexanes (1:2, v/v) mixture. The data crystal had approximate dimensions; 0.22 x 0.081 x 0.024 mm. The data were collected on an Agilent Technologies SuperNova Dual Source diffractometer using a μ-focus Cu Kα radiation source (λ = 1.5418 Å) with collimating mirror monochromators. A total of 1337 frames of data were collected using ω-scans with a scan range of 1° and a counting time of 10.5 seconds per frame for frames collected with a detector offset of +/- 41.6° and 32 seconds per frame with frames collected with a detector offset of 112.0°. The data were collected at 100 K using an Oxford Cryostream low temperature device.Details of crystal data, data collection and structure refinement are listed in Table S1. Data collection, unit cell refinement and data reduction were performed using Rigaku Oxford Diffraction's CrysAlisPro V 1.171.40.53.[1] The structure was solved by direct methods using SHELXT[2] and refined by full-matrix least-squares on F^2 with anisotropic displacement parameters for the non-H atoms using SHELXL-2016/6.[3] Structure analysis was aided by use of the programs PLATON[4], OLEX2[5] and WinGX.[6] The hydrogen atoms on the carbon atoms were calculated in ideal positions with isotropic displacement parameters set to 1.2xUeq of the attached atom (1.5xUeq for methyl hydrogen atoms).

There are several areas of disorder present in the crystal. A molecule of dichloromethane is disordered about two orientations. The disorder for this molecule was modeled using features present in OLEX2. The pyrihexaphyrin macrocycle is bound to two Cu(II) ions. The Cu coordination is completed by coordination by two acetate ions above and below the plane through the macrocycle. Coordination by the acetate ions is through a bridging carbonyl oxygen atom. One of the acetate ions was disordered (C59, C60, O3 and O4). The disorder involved a small rotation about the carbonyl oxygen (O3) to carbon bond C59) where the carbonyl oxygen atom was coordinated to the two Cu ions. Finally, there appeared to be a mixture of an acetate ion and a chloride ion bound to the Cu ions. In the original model, displacement parameters for the carbonyl group, O1 and C57, were elongated towards each other resulting in a Hirshfeld cif alert. Once these atoms were refined isotropically, a large peak (~2.5e-/A^3) was observed between O1 and C57. The contact distance to the Cu(II) ions was consistent with an expected Cl...Cu contact. Chloride ion was a possible contaminant in the solution and it seemed reasonable to assign this extra peak as a low occupancy chloride ion. To maintain charge neutrality, the site occupancy factors for the acetate ion and the chloride ion were fixed to be unity. The site occupancy factors were refined while constraining the isotropic displacement parameters for the two ions to be equal. The site occupancy factor for the chloride ion refined to 11%.

The function, $\Sigma w(|F_o|^2 - |F_c|^2)^2$, was minimized, where $w = 1/[(\square(F_o))^2 + (0.0804*P)^2 + (3.3748*P)]$ and $P = (|F_o|^2 + 2|F_c|^2)/3$. $R_w(F^2)$ refined to 0.152, with R(F) equal to 0.0551 and a goodness of fit, S, = 1.02. Definitions used for calculating R(F), $R_w(F^2)$ and the goodness of fit, S, are given below.[7] The data were checked for secondary extinction effects, but no correction was necessary. Neutral atom scattering factors and values used to calculate the linear absorption coefficient are from the International Tables for X-ray Crystallography (1992).[8] All figures were generated using SHELXTL/PC.[9] Tables of positional and thermal parameters, bond lengths and angles, torsion angles and figures are found below.

Table 1. Crystal data and structure refinement for **2**.

Identification code	jtb-dpam-biscu-x2-redo	
Empirical formula	C61 H65 Cl2 Cu2 N6 O4.50	
Formula weight	1152.17	
Temperature	100.15 K	
Wavelength	1.54184 Å	
Crystal system	triclinic	
Space group	P -1	
Unit cell dimensions	a = 12.7793(4) Å	α = 112.327(3)°.
	b = 14.4880(6) Å	β = 95.763(3)°.
	c = 17.7112(5) Å	γ = 110.276(3)°.
Volume	2741.73(17) $Å^3$	
Z	2	
Density (calculated)	1.396 Mg/m^3	
Absorption coefficient	2.287 mm^{-1}	
F(000)	1202	
Crystal size	0.22 x 0.081 x 0.024 mm^3	
Theta range for data collection	2.799 to 68.244°.	
Index ranges	-15<=h<=15, -17<=k<=15, -16<=l<=21	
Reflections collected	20493	
Independent reflections	10031 [R(int) = 0.0382]	
Completeness to theta = 67.684°	99.8 %	
Absorption correction	Gaussian and multi-scan	
Max. and min. transmission	1.00 and 0.631	
Refinement method	Full-matrix least-squares on F^2	
Data / restraints / parameters	10031 / 120 / 742	
Goodness-of-fit on F^2	1.022	
Final R indices [I>2sigma(I)]	R1 = 0.0551, wR2 = 0.1421	
R indices (all data)	R1 = 0.0668, wR2 = 0.1524	
Extinction coefficient	n/a	
Largest diff. peak and hole	1.631 and -1.096 e.$Å^{-3}$	

Table 2. Atomic coordinates (x 10^4) and equivalent isotropic displacement parameters ($Å^2$ x 10^3) for **2**. U(eq) is defined as one third of the trace of the orthogonalized U^{ij} tensor.

	x	y	z	U(eq)
C1	5031(2)	4113(2)	1070(2)	16(1)
C2	5547(2)	3356(2)	967(2)	17(1)
C3	4671(3)	2315(2)	524(2)	18(1)
C4	3620(3)	2454(2)	352(2)	18(1)
C5	2513(3)	1642(2)	-157(2)	20(1)
C6	1496(3)	1827(3)	-183(2)	22(1)
C7	384(3)	1248(3)	-793(2)	26(1)
C8	-231(3)	1878(3)	-516(2)	26(1)
C9	515(3)	2820(3)	251(2)	24(1)
C10	441(2)	3813(3)	858(2)	22(1)
C11	-427(3)	4147(3)	725(2)	26(1)
C12	-356(3)	5120(3)	1339(2)	28(1)
C13	512(3)	5691(3)	2089(2)	24(1)
C14	1361(2)	5317(3)	2202(2)	20(1)
C15	2177(2)	5916(2)	3073(2)	18(1)
C16	1736(3)	6043(3)	3805(2)	20(1)
C17	2659(3)	6782(2)	4501(2)	19(1)
C18	3659(2)	7115(2)	4186(2)	17(1)
C19	4772(2)	7944(2)	4638(2)	16(1)
C20	5727(2)	8207(2)	4286(2)	18(1)
C21	6877(2)	9089(2)	4616(2)	19(1)
C22	7428(2)	8878(2)	3968(2)	18(1)
C23	6628(2)	7878(2)	3279(2)	18(1)
C24	6672(2)	7124(2)	2463(2)	16(1)
C25	7486(2)	7418(2)	2032(2)	19(1)
C26	7409(3)	6641(3)	1252(2)	21(1)
C27	6548(3)	5584(2)	930(2)	20(1)
C28	5777(2)	5317(2)	1399(2)	16(1)
C29	6804(3)	3660(3)	1309(2)	23(1)
C30	7205(3)	4238(4)	2267(2)	42(1)
C31	4816(3)	1282(2)	395(2)	23(1)

C32	4496(3)	941(3)	1091(2)	31(1)
C33	2424(3)	540(2)	-731(2)	22(1)
C34	2952(3)	410(2)	-1390(2)	22(1)
C35	2876(3)	-619(3)	-1926(2)	27(1)
C36	2295(3)	-1521(3)	-1800(2)	33(1)
C37	1771(3)	-1412(3)	-1148(2)	36(1)
C38	1817(3)	-391(3)	-618(2)	31(1)
C39	-58(3)	191(3)	-1606(2)	34(1)
C40	-868(3)	-830(3)	-1532(3)	46(1)
C41	-1425(3)	1629(3)	-980(2)	32(1)
C42	-1370(3)	2196(3)	-1551(2)	38(1)
C43	512(3)	5399(3)	3792(2)	28(1)
C44	203(3)	4180(3)	3468(3)	43(1)
C45	2581(3)	7050(3)	5398(2)	29(1)
C46	2814(4)	6259(4)	5687(3)	47(1)
C47	4999(2)	8654(2)	5557(2)	19(1)
C48	4641(3)	9504(3)	5813(2)	23(1)
C49	4894(3)	10198(3)	6666(2)	28(1)
C50	5504(3)	10035(3)	7264(2)	30(1)
C51	5854(3)	9195(3)	7015(2)	27(1)
C52	5612(3)	8502(3)	6161(2)	21(1)
C53	7463(3)	10071(3)	5470(2)	24(1)
C54	8287(3)	9902(3)	6046(2)	32(1)
C55	8632(2)	9596(3)	4006(2)	21(1)
C56	8654(3)	10405(3)	3645(2)	31(1)
C59	4201(3)	4260(3)	2899(2)	32(1)
N1	3887(2)	3590(2)	735(2)	17(1)
N2	1515(2)	2753(2)	436(2)	20(1)
N3	1353(2)	4422(2)	1572(2)	19(1)
N4	3312(2)	6516(2)	3297(2)	17(1)
N5	5636(2)	7516(2)	3479(2)	18(1)
N6	5823(2)	6085(2)	2144(2)	15(1)
Cu1	2738(1)	4110(1)	1204(1)	22(1)
Cu2	4483(1)	6127(1)	2740(1)	18(1)
O1	3572(3)	5664(3)	1455(2)	18(1)
O2	2303(2)	5607(2)	454(2)	27(1)

C57	3226(3)	6099(3)	1019(3)	20(1)
C58	4031(4)	7246(3)	1241(3)	42(1)
O3	3670(2)	4558(2)	2449(1)	18(1)
O4	5147(9)	4820(15)	3434(9)	55(1)
C60	3491(13)	3091(8)	2681(16)	29(2)
O4A	5016(5)	4961(6)	3534(4)	55(1)
C60A	3820(5)	3057(4)	2637(6)	29(2)
Cl1	1206(6)	2869(6)	5692(4)	68(2)
Cl2	2419(4)	3183(3)	4441(2)	71(1)
C1A	2530(15)	3240(19)	5389(13)	42(3)
Cl1A	1147(8)	2832(7)	5874(5)	88(2)
Cl2A	1688(4)	2896(3)	4348(3)	100(2)
C1B	2263(16)	3200(20)	5483(14)	50(4)
O1W	5380(9)	5971(6)	5217(5)	77(2)
Cl3	3429(9)	5943(10)	1299(6)	25(3)

Table 3. Bond lengths [Å] and angles [°] for **2**.

C1-C2	1.430(4)	C17-C45	1.509(4)
C1-C28	1.501(4)	C18-C19	1.402(4)
C1-N1	1.334(4)	C18-N4	1.408(4)
C2-C3	1.377(4)	C19-C20	1.426(4)
C2-C29	1.501(4)	C19-C47	1.489(4)
C3-C4	1.445(4)	C20-C21	1.447(4)
C3-C31	1.506(4)	C20-N5	1.363(4)
C4-C5	1.409(4)	C21-C22	1.397(4)
C4-N1	1.415(4)	C21-C53	1.507(4)
C5-C6	1.414(5)	C22-C23	1.418(4)
C5-C33	1.489(4)	C22-C55	1.507(4)
C6-C7	1.438(4)	C23-C24	1.465(4)
C6-N2	1.363(4)	C23-N5	1.333(4)
C7-C8	1.387(5)	C24-C25	1.394(4)
C7-C39	1.517(5)	C24-N6	1.364(4)
C8-C9	1.431(4)	C25-H25	0.95
C8-C41	1.508(4)	C25-C26	1.380(4)
C9-C10	1.474(5)	C26-H26	0.95
C9-N2	1.333(4)	C26-C27	1.388(4)
C10-C11	1.388(5)	C27-H27	0.95
C10-N3	1.370(4)	C27-C28	1.397(4)
C11-H11	0.95	C28-N6	1.344(4)
C11-C12	1.381(5)	C29-H29A	0.99
C12-H12	0.95	C29-H29B	0.99
C12-C13	1.383(5)	C29-C30	1.512(5)
C13-H13	0.95	C30-H30A	0.98
C13-C14	1.399(4)	C30-H30B	0.98
C14-C15	1.501(4)	C30-H30C	0.98
C14-N3	1.347(4)	C31-H31A	0.99
C15-C16	1.438(4)	C31-H31B	0.99
C15-N4	1.328(4)	C31-C32	1.534(4)
C16-C17	1.374(4)	C32-H32A	0.98
C16-C43	1.511(4)	C32-H32B	0.98
C17-C18	1.447(4)	C32-H32C	0.98

C33-C34	1.390(5)	C47-C52	1.393(4)
C33-C38	1.404(4)	C48-H48	0.95
C34-H34	0.95	C48-C49	1.385(5)
C34-C35	1.393(5)	C49-H49	0.95
C35-H35	0.95	C49-C50	1.392(5)
C35-C36	1.375(5)	C50-H50	0.95
C36-H36	0.95	C50-C51	1.372(5)
C36-C37	1.378(6)	C51-H51	0.95
C37-H37	0.95	C51-C52	1.388(5)
C37-C38	1.395(5)	C52-H52	0.95
C38-H38	0.95	C53-H53A	0.99
C39-H39A	0.99	C53-H53B	0.99
C39-H39B	0.99	C53-C54	1.538(5)
C39-C40	1.547(5)	C54-H54A	0.98
C40-H40A	0.98	C54-H54B	0.98
C40-H40B	0.98	C54-H54C	0.98
C40-H40C	0.98	C55-H55A	0.99
C41-H41A	0.99	C55-H55B	0.99
C41-H41B	0.99	C55-C56	1.527(5)
C41-C42	1.520(5)	C56-H56A	0.98
C42-H42A	0.98	C56-H56B	0.98
C42-H42B	0.98	C56-H56C	0.98
C42-H42C	0.98	C59-O3	1.280(4)
C43-H43A	0.99	C59-O4	1.235(8)
C43-H43B	0.99	C59-C60	1.486(8)
C43-C44	1.519(5)	C59-O4A	1.243(5)
C44-H44A	0.98	C59-C60A	1.497(5)
C44-H44B	0.98	N1-Cu1	1.987(3)
C44-H44C	0.98	N2-Cu1	1.889(2)
C45-H45A	0.99	N3-Cu1	2.080(2)
C45-H45B	0.99	N4-Cu2	1.998(2)
C45-C46	1.522(6)	N5-Cu2	1.867(2)
C46-H46A	0.98	N6-Cu2	2.107(2)
C46-H46B	0.98	Cu1-Cu2	3.0237(6)
C46-H46C	0.98	Cu1-O1	1.971(4)
C47-C48	1.392(4)	Cu1-O3	2.134(2)

Cu1-Cl3	2.422(13)	C60A-H60D	0.98
Cu2-O1	2.181(4)	C60A-H60E	0.98
Cu2-O3	1.968(2)	C60A-H60F	0.98
Cu2-Cl3	2.636(14)	Cl1-C1A	1.792(17)
O1-C57	1.297(7)	Cl2-C1A	1.64(2)
O2-C57	1.240(5)	C1A-H1AA	0.99
C57-C58	1.486(5)	C1A-H1AB	0.99
C58-H58A	0.98	Cl1A-C1B	1.66(2)
C58-H58B	0.98	Cl2A-C1B	1.89(2)
C58-H58C	0.98	C1B-H1BA	0.99
C60-H60A	0.98	C1B-H1BB	0.99
C60-H60B	0.98	O1W-H1WA	0.8389
C60-H60C	0.98	O1W-H1WB	0.8412

C2-C1-C28	120.4(2)	C7-C8-C41	125.5(3)
N1-C1-C2	111.4(3)	C9-C8-C41	127.8(3)
N1-C1-C28	127.6(3)	C8-C9-C10	135.9(3)
C1-C2-C29	125.5(3)	N2-C9-C8	109.4(3)
C3-C2-C1	107.1(3)	N2-C9-C10	114.7(3)
C3-C2-C29	127.4(3)	C11-C10-C9	124.7(3)
C2-C3-C4	106.4(3)	N3-C10-C9	113.4(3)
C2-C3-C31	123.9(3)	N3-C10-C11	121.8(3)
C4-C3-C31	129.1(3)	C10-C11-H11	120.9
C5-C4-C3	128.2(3)	C12-C11-C10	118.1(3)
C5-C4-N1	123.2(3)	C12-C11-H11	120.9
N1-C4-C3	108.5(2)	C11-C12-H12	120.0
C4-C5-C6	124.0(3)	C11-C12-C13	120.1(3)
C4-C5-C33	117.6(3)	C13-C12-H12	120.0
C6-C5-C33	118.3(3)	C12-C13-H13	120.1
C5-C6-C7	132.5(3)	C12-C13-C14	119.8(3)
N2-C6-C5	119.0(3)	C14-C13-H13	120.1
N2-C6-C7	108.3(3)	C13-C14-C15	115.4(3)
C6-C7-C39	128.9(3)	N3-C14-C13	120.1(3)
C8-C7-C6	106.3(3)	N3-C14-C15	124.3(3)
C8-C7-C39	124.7(3)	C16-C15-C14	120.3(3)
C7-C8-C9	106.6(3)	N4-C15-C14	127.8(3)

N4-C15-C16	111.1(3)	C28-C27-H27	120.0
C15-C16-C43	125.5(3)	C27-C28-C1	115.2(3)
C17-C16-C15	106.7(3)	N6-C28-C1	123.5(2)
C17-C16-C43	127.7(3)	N6-C28-C27	120.8(3)
C16-C17-C18	106.8(3)	C2-C29-H29A	108.9
C16-C17-C45	123.1(3)	C2-C29-H29B	108.9
C18-C17-C45	129.9(3)	C2-C29-C30	113.2(3)
C19-C18-C17	128.6(3)	H29A-C29-H29B	107.7
C19-C18-N4	123.1(3)	C30-C29-H29A	108.9
N4-C18-C17	108.1(2)	C30-C29-H29B	108.9
C18-C19-C20	125.4(3)	C29-C30-H30A	109.5
C18-C19-C47	119.0(3)	C29-C30-H30B	109.5
C20-C19-C47	115.6(3)	C29-C30-H30C	109.5
C19-C20-C21	133.4(3)	H30A-C30-H30B	109.5
N5-C20-C19	119.1(3)	H30A-C30-H30C	109.5
N5-C20-C21	107.5(3)	H30B-C30-H30C	109.5
C20-C21-C53	131.0(3)	C3-C31-H31A	109.4
C22-C21-C20	106.4(3)	C3-C31-H31B	109.4
C22-C21-C53	122.7(3)	C3-C31-C32	111.0(3)
C21-C22-C23	106.5(3)	H31A-C31-H31B	108.0
C21-C22-C55	125.8(3)	C32-C31-H31A	109.4
C23-C22-C55	127.7(3)	C32-C31-H31B	109.4
C22-C23-C24	135.6(3)	C31-C32-H32A	109.5
N5-C23-C22	109.7(3)	C31-C32-H32B	109.5
N5-C23-C24	114.4(3)	C31-C32-H32C	109.5
C25-C24-C23	124.4(3)	H32A-C32-H32B	109.5
N6-C24-C23	113.7(2)	H32A-C32-H32C	109.5
N6-C24-C25	121.9(3)	H32B-C32-H32C	109.5
C24-C25-H25	120.6	C34-C33-C5	120.5(3)
C26-C25-C24	118.9(3)	C34-C33-C38	118.4(3)
C26-C25-H25	120.6	C38-C33-C5	121.1(3)
C25-C26-H26	120.5	C33-C34-H34	119.6
C25-C26-C27	119.1(3)	C33-C34-C35	120.7(3)
C27-C26-H26	120.5	C35-C34-H34	119.6
C26-C27-H27	120.0	C34-C35-H35	119.8
C26-C27-C28	120.0(3)	C36-C35-C34	120.3(3)

C36-C35-H35	119.8	C16-C43-C44	112.7(3)
C35-C36-H36	120.0	H43A-C43-H43B	107.8
C35-C36-C37	120.0(3)	C44-C43-H43A	109.1
C37-C36-H36	120.0	C44-C43-H43B	109.1
C36-C37-H37	119.9	C43-C44-H44A	109.5
C36-C37-C38	120.3(3)	C43-C44-H44B	109.5
C38-C37-H37	119.9	C43-C44-H44C	109.5
C33-C38-H38	119.9	H44A-C44-H44B	109.5
C37-C38-C33	120.3(3)	H44A-C44-H44C	109.5
C37-C38-H38	119.9	H44B-C44-H44C	109.5
C7-C39-H39A	108.8	C17-C45-H45A	109.4
C7-C39-H39B	108.8	C17-C45-H45B	109.4
C7-C39-C40	113.8(3)	C17-C45-C46	111.2(3)
H39A-C39-H39B	107.7	H45A-C45-H45B	108.0
C40-C39-H39A	108.8	C46-C45-H45A	109.4
C40-C39-H39B	108.8	C46-C45-H45B	109.4
C39-C40-H40A	109.5	C45-C46-H46A	109.5
C39-C40-H40B	109.5	C45-C46-H46B	109.5
C39-C40-H40C	109.5	C45-C46-H46C	109.5
H40A-C40-H40B	109.5	H46A-C46-H46B	109.5
H40A-C40-H40C	109.5	H46A-C46-H46C	109.5
H40B-C40-H40C	109.5	H46B-C46-H46C	109.5
C8-C41-H41A	109.3	C48-C47-C19	120.0(3)
C8-C41-H41B	109.3	C48-C47-C52	119.8(3)
C8-C41-C42	111.5(3)	C52-C47-C19	120.2(3)
H41A-C41-H41B	108.0	C47-C48-H48	119.9
C42-C41-H41A	109.3	C49-C48-C47	120.3(3)
C42-C41-H41B	109.3	C49-C48-H48	119.9
C41-C42-H42A	109.5	C48-C49-H49	120.3
C41-C42-H42B	109.5	C48-C49-C50	119.4(3)
C41-C42-H42C	109.5	C50-C49-H49	120.3
H42A-C42-H42B	109.5	C49-C50-H50	119.7
H42A-C42-H42C	109.5	C51-C50-C49	120.6(3)
H42B-C42-H42C	109.5	C51-C50-H50	119.7
C16-C43-H43A	109.1	C50-C51-H51	119.8
C16-C43-H43B	109.1	C50-C51-C52	120.3(3)

C52-C51-H51	119.8	C4-N1-Cu1	120.07(19)
C47-C52-H52	120.2	C6-N2-Cu1	132.4(2)
C51-C52-C47	119.6(3)	C9-N2-C6	109.3(3)
C51-C52-H52	120.2	C9-N2-Cu1	113.7(2)
C21-C53-H53A	109.2	C10-N3-Cu1	105.6(2)
C21-C53-H53B	109.2	C14-N3-C10	119.6(3)
C21-C53-C54	112.3(3)	C14-N3-Cu1	129.28(19)
H53A-C53-H53B	107.9	C15-N4-C18	107.3(2)
C54-C53-H53A	109.2	C15-N4-Cu2	127.5(2)
C54-C53-H53B	109.1	C18-N4-Cu2	116.87(18)
C53-C54-H54A	109.5	C20-N5-Cu2	130.3(2)
C53-C54-H54B	109.5	C23-N5-C20	110.0(2)
C53-C54-H54C	109.5	C23-N5-Cu2	118.9(2)
H54A-C54-H54B	109.5	C24-N6-Cu2	108.95(18)
H54A-C54-H54C	109.5	C28-N6-C24	119.2(2)
H54B-C54-H54C	109.5	C28-N6-Cu2	128.51(19)
C22-C55-H55A	108.9	N1-Cu1-N3	171.54(10)
C22-C55-H55B	108.9	N1-Cu1-Cu2	95.75(7)
C22-C55-C56	113.2(3)	N1-Cu1-O3	89.70(9)
H55A-C55-H55B	107.8	N1-Cu1-Cl3	102.4(3)
C56-C55-H55A	108.9	N2-Cu1-N1	90.49(11)
C56-C55-H55B	108.9	N2-Cu1-N3	81.07(11)
C55-C56-H56A	109.5	N2-Cu1-Cu2	166.54(8)
C55-C56-H56B	109.5	N2-Cu1-O1	144.78(14)
C55-C56-H56C	109.5	N2-Cu1-O3	128.01(9)
H56A-C56-H56B	109.5	N2-Cu1-Cl3	133.5(3)
H56A-C56-H56C	109.5	N3-Cu1-Cu2	92.39(7)
H56B-C56-H56C	109.5	N3-Cu1-O3	95.06(9)
O3-C59-C60	109.0(9)	N3-Cu1-Cl3	84.0(3)
O3-C59-C60A	118.4(4)	O1-Cu1-N1	97.44(12)
O4-C59-O3	127.5(12)	O1-Cu1-N3	89.84(12)
O4-C59-C60	123.4(10)	O1-Cu1-Cu2	46.07(12)
O4A-C59-O3	120.1(6)	O1-Cu1-O3	86.48(13)
O4A-C59-C60A	121.4(5)	O3-Cu1-Cu2	40.43(5)
C1-N1-C4	106.6(2)	O3-Cu1-Cl3	97.0(3)
C1-N1-Cu1	125.1(2)	Cl3-Cu1-Cu2	56.6(3)

N4-Cu2-N6	166.06(10)	H58B-C58-H58C	109.5
N4-Cu2-Cu1	95.54(7)	C59-O3-Cu1	144.76(19)
N4-Cu2-O1	96.43(11)	C59-O3-Cu2	112.54(19)
N4-Cu2-Cl3	91.4(2)	Cu2-O3-Cu1	94.89(8)
N5-Cu2-N4	90.11(11)	C59-C60-H60A	109.5
N5-Cu2-N6	80.87(10)	C59-C60-H60B	109.5
N5-Cu2-Cu1	164.80(8)	C59-C60-H60C	109.5
N5-Cu2-O1	124.79(13)	H60A-C60-H60B	109.5
N5-Cu2-O3	149.21(10)	H60A-C60-H60C	109.5
N5-Cu2-Cl3	115.8(3)	H60B-C60-H60C	109.5
N6-Cu2-Cu1	90.75(7)	C59-C60A-H60D	109.5
N6-Cu2-O1	80.33(11)	C59-C60A-H60E	109.5
N6-Cu2-Cl3	83.1(2)	C59-C60A-H60F	109.5
O1-Cu2-Cu1	40.59(10)	H60D-C60A-H60E	109.5
O3-Cu2-N4	93.27(9)	H60D-C60A-H60F	109.5
O3-Cu2-N6	99.93(9)	H60E-C60A-H60F	109.5
O3-Cu2-Cu1	44.67(6)	Cl1-C1A-H1AA	108.2
O3-Cu2-O1	85.24(12)	Cl1-C1A-H1AB	108.2
O3-Cu2-Cl3	94.7(3)	Cl2-C1A-Cl1	116.2(12)
Cl3-Cu2-Cu1	50.1(3)	Cl2-C1A-H1AA	108.2
Cu1-O1-Cu2	93.34(18)	Cl2-C1A-H1AB	108.2
C57-O1-Cu1	122.5(3)	H1AA-C1A-H1AB	107.4
C57-O1-Cu2	139.7(3)	Cl1A-C1B-Cl2A	108.4(11)
O1-C57-C58	114.8(4)	Cl1A-C1B-H1BA	110.0
O2-C57-O1	123.4(4)	Cl1A-C1B-H1BB	110.0
O2-C57-C58	121.8(5)	Cl2A-C1B-H1BA	110.0
C57-C58-H58A	109.5	Cl2A-C1B-H1BB	110.0
C57-C58-H58B	109.5	H1BA-C1B-H1BB	108.4
C57-C58-H58C	109.5	H1WA-O1W-H1WB	130.4
H58A-C58-H58B	109.5	Cu1-Cl3-Cu2	73.3(4)
H58A-C58-H58C	109.5		

Table 4. Anisotropic displacement parameters ($Å^2$x 10^3) for **2**. The anisotropic displacement factor exponent takes the form: $-2\pi^2[h^2 a^{*2}U^{11} + ... + 2 h k a^* b^* U^{12}]$

	U^{11}	U^{22}	U^{33}	U^{23}	U^{13}	U^{12}
C1	14(1)	20(1)	13(1)	6(1)	5(1)	5(1)
C2	17(1)	19(1)	14(1)	8(1)	6(1)	6(1)
C3	18(1)	20(1)	15(1)	8(1)	6(1)	5(1)
C4	20(1)	16(1)	14(1)	6(1)	4(1)	3(1)
C5	20(2)	21(2)	16(1)	11(1)	4(1)	2(1)
C6	18(2)	22(2)	17(1)	7(1)	4(1)	0(1)
C7	16(1)	33(2)	20(2)	11(1)	4(1)	1(1)
C8	13(1)	33(2)	21(2)	10(1)	2(1)	1(1)
C9	13(1)	33(2)	19(1)	12(1)	3(1)	2(1)
C10	10(1)	35(2)	21(1)	15(1)	5(1)	5(1)
C11	11(1)	40(2)	25(2)	17(1)	4(1)	6(1)
C12	14(1)	40(2)	35(2)	23(2)	7(1)	11(1)
C13	16(1)	34(2)	28(2)	16(1)	7(1)	12(1)
C14	11(1)	29(2)	23(2)	15(1)	7(1)	7(1)
C15	12(1)	23(1)	20(1)	9(1)	5(1)	9(1)
C16	14(1)	25(2)	22(2)	12(1)	8(1)	9(1)
C17	18(1)	23(2)	22(2)	12(1)	8(1)	12(1)
C18	15(1)	21(1)	20(1)	10(1)	6(1)	10(1)
C19	17(1)	20(1)	17(1)	10(1)	5(1)	11(1)
C20	14(1)	20(1)	17(1)	7(1)	3(1)	8(1)
C21	14(1)	21(1)	20(1)	9(1)	2(1)	8(1)
C22	13(1)	20(1)	20(1)	8(1)	4(1)	6(1)
C23	16(1)	20(1)	20(1)	9(1)	6(1)	10(1)
C24	10(1)	17(1)	18(1)	7(1)	2(1)	5(1)
C25	14(1)	19(1)	26(2)	11(1)	8(1)	4(1)
C26	17(1)	25(2)	24(2)	14(1)	10(1)	7(1)
C27	19(1)	22(2)	17(1)	7(1)	7(1)	6(1)
C28	11(1)	20(1)	18(1)	9(1)	4(1)	6(1)
C29	16(1)	22(2)	31(2)	11(1)	7(1)	9(1)
C30	27(2)	59(3)	32(2)	11(2)	-2(2)	23(2)
C31	26(2)	19(1)	20(1)	7(1)	3(1)	7(1)

C32	35(2)	33(2)	34(2)	22(2)	13(2)	16(2)
C33	22(2)	17(1)	18(1)	6(1)	0(1)	0(1)
C34	19(1)	20(1)	21(1)	11(1)	1(1)	3(1)
C35	26(2)	27(2)	20(1)	8(1)	1(1)	8(1)
C36	38(2)	19(2)	28(2)	5(1)	2(2)	5(1)
C37	40(2)	22(2)	37(2)	14(2)	8(2)	2(2)
C38	32(2)	24(2)	27(2)	13(1)	10(1)	-1(1)
C39	20(2)	38(2)	26(2)	3(2)	2(1)	5(1)
C40	26(2)	29(2)	52(2)	1(2)	8(2)	-2(2)
C41	19(2)	38(2)	26(2)	10(2)	-1(1)	6(1)
C42	34(2)	43(2)	32(2)	18(2)	2(2)	14(2)
C43	14(1)	41(2)	24(2)	13(1)	9(1)	8(1)
C44	25(2)	36(2)	55(2)	17(2)	15(2)	1(2)
C45	21(2)	34(2)	22(2)	9(1)	9(1)	4(1)
C46	55(3)	56(3)	36(2)	32(2)	11(2)	16(2)
C47	14(1)	21(1)	18(1)	8(1)	6(1)	6(1)
C48	21(2)	27(2)	23(2)	12(1)	6(1)	11(1)
C49	29(2)	25(2)	27(2)	8(1)	10(1)	12(1)
C50	31(2)	31(2)	18(2)	6(1)	7(1)	9(1)
C51	24(2)	35(2)	22(2)	14(1)	5(1)	11(1)
C52	20(1)	26(2)	22(2)	14(1)	7(1)	11(1)
C53	18(1)	24(2)	20(2)	5(1)	4(1)	5(1)
C54	20(2)	46(2)	21(2)	9(2)	3(1)	10(2)
C55	13(1)	23(2)	21(1)	6(1)	4(1)	4(1)
C56	28(2)	27(2)	32(2)	15(2)	6(1)	4(1)
C59	40(2)	32(2)	23(2)	14(2)	4(2)	12(2)
N1	14(1)	18(1)	15(1)	6(1)	4(1)	4(1)
N2	11(1)	25(1)	18(1)	10(1)	2(1)	1(1)
N3	9(1)	30(1)	18(1)	12(1)	4(1)	7(1)
N4	13(1)	23(1)	18(1)	10(1)	4(1)	9(1)
N5	13(1)	20(1)	19(1)	7(1)	5(1)	6(1)
N6	9(1)	20(1)	17(1)	9(1)	5(1)	7(1)
Cu1	10(1)	26(1)	21(1)	4(1)	1(1)	5(1)
Cu2	11(1)	19(1)	19(1)	4(1)	6(1)	3(1)
O1	20(1)	19(2)	18(2)	12(1)	2(1)	6(1)
O2	24(1)	44(2)	21(1)	16(1)	5(1)	20(1)

C57	28(2)	25(2)	19(2)	15(2)	15(2)	16(2)
C58	55(3)	24(2)	38(2)	14(2)	0(2)	10(2)
O3	14(1)	19(1)	17(1)	7(1)	1(1)	3(1)
O4	63(2)	30(3)	47(2)	12(2)	-27(2)	10(2)
C60	24(4)	30(2)	42(2)	20(2)	13(3)	15(2)
O4A	63(2)	30(3)	47(2)	12(2)	-27(2)	10(2)
C60A	24(4)	30(2)	42(2)	20(2)	13(3)	15(2)
Cl1	43(2)	73(3)	66(2)	6(2)	14(1)	29(2)
Cl2	115(3)	81(2)	46(1)	30(1)	19(2)	72(2)
C1A	39(6)	51(4)	48(5)	25(4)	21(4)	25(5)
Cl1A	70(3)	61(3)	156(6)	51(4)	40(4)	48(2)
Cl2A	112(3)	62(2)	97(3)	44(2)	-35(3)	11(2)
C1B	52(8)	52(5)	46(6)	28(4)	2(5)	20(6)
O1W	115(7)	66(5)	45(4)	25(4)	19(4)	35(5)
Cl3	30(4)	34(5)	13(4)	16(3)	13(3)	8(3)

Table 5. Hydrogen coordinates (x 10^4) and isotropic displacement parameters (Å^2 x 10^3) for **2**.

	x	y	z	U(eq)
H11	-1052	3718	226	31
H12	-904	5398	1246	33
H13	532	6335	2527	29
H25	8083	8141	2272	23
H26	7939	6827	938	25
H27	6482	5041	392	24
H29A	7274	4145	1085	28
H29B	6942	2986	1105	28
H30A	7104	4925	2473	62
H30B	8024	4402	2452	62
H30C	6745	3763	2494	62
H31A	5630	1406	402	27
H31B	4316	683	-165	27
H32A	4981	1539	1646	46
H32B	4621	282	1005	46
H32C	3679	784	1066	46
H34	3369	1030	-1476	26
H35	3227	-698	-2381	32
H36	2255	-2219	-2163	40
H37	1376	-2035	-1059	43
H38	1437	-325	-178	37
H39A	613	67	-1780	41
H39B	-480	277	-2058	41
H40A	-468	-908	-1074	68
H40B	-1083	-1487	-2067	68
H40C	-1568	-743	-1409	68
H41A	-1849	1876	-563	38
H41B	-1856	825	-1326	38
H42A	-1064	2991	-1203	56
H42B	-2149	1930	-1907	56
H42C	-862	2033	-1912	56

H43A	-32	5502	3426	33
H43B	418	5692	4372	33
H44A	265	3878	2885	65
H44B	-592	3802	3481	65
H44C	736	4072	3829	65
H45A	1799	7009	5433	34
H45B	3151	7810	5780	34
H46A	2222	5512	5330	71
H46B	2790	6471	6278	71
H46C	3580	6285	5639	71
H48	4221	9607	5401	27
H49	4653	10780	6841	33
H50	5680	10509	7849	35
H51	6264	9088	7429	32
H52	5863	7926	5991	26
H53A	6865	10216	5750	29
H53B	7905	10724	5390	29
H54A	7841	9303	6174	49
H54B	8694	10580	6575	49
H54C	8852	9716	5758	49
H55A	8994	9124	3686	26
H55B	9102	10013	4603	26
H56A	8197	9998	3052	47
H56B	9455	10841	3680	47
H56C	8323	10893	3972	47
H58A	4207	7700	1851	63
H58B	3667	7538	931	63
H58C	4748	7254	1087	63
H60A	2750	3016	2817	44
H60B	3905	2849	3009	44
H60C	3350	2638	2075	44
H60D	3151	2792	2846	44
H60E	4455	2933	2875	44
H60F	3605	2660	2017	44
H1AA	3061	3999	5807	50
H1AB	2893	2751	5426	50

H1BA	2730	2785	5505	60
H1BB	2763	3994	5822	60
H1WA	5161	5611	4687	115
H1WB	5432	5761	5596	115

Table S6. Torsion angles [°] for **2**.

C1-C2-C3-C4	0.7(3)	C6-C7-C39-C40	101.6(4)
C1-C2-C3-C31	-170.5(3)	C6-N2-Cu1-N1	-0.7(3)
C1-C2-C29-C30	63.5(4)	C6-N2-Cu1-N3	179.9(3)
C1-C28-N6-C24	168.1(3)	C6-N2-Cu1-Cu2	-118.4(4)
C1-C28-N6-Cu2	-34.8(4)	C6-N2-Cu1-O1	103.0(3)
C2-C1-C28-C27	57.2(4)	C6-N2-Cu1-O3	-90.7(3)
C2-C1-C28-N6	-114.7(3)	C6-N2-Cu1-Cl3	107.0(4)
C2-C1-N1-C4	-3.4(3)	C7-C6-N2-C9	-1.9(3)
C2-C1-N1-Cu1	144.6(2)	C7-C6-N2-Cu1	-155.7(2)
C2-C3-C4-C5	172.8(3)	C7-C8-C9-C10	178.4(3)
C2-C3-C4-N1	-2.7(3)	C7-C8-C9-N2	-1.2(4)
C2-C3-C31-C32	92.6(4)	C7-C8-C41-C42	-92.4(4)
C3-C2-C29-C30	-114.2(4)	C8-C7-C39-C40	-81.6(5)
C3-C4-C5-C6	169.6(3)	C8-C9-C10-C11	-10.7(6)
C3-C4-C5-C33	-13.5(4)	C8-C9-C10-N3	171.8(3)
C3-C4-N1-C1	3.7(3)	C8-C9-N2-C6	1.9(3)
C3-C4-N1-Cu1	-146.22(19)	C8-C9-N2-Cu1	161.1(2)
C4-C3-C31-C32	-76.4(4)	C9-C8-C41-C42	83.3(4)
C4-C5-C6-C7	160.1(3)	C9-C10-C11-C12	-177.5(3)
C4-C5-C6-N2	-14.7(4)	C9-C10-N3-C14	-176.1(3)
C4-C5-C33-C34	-64.3(4)	C9-C10-N3-Cu1	27.7(3)
C4-C5-C33-C38	115.2(3)	C9-N2-Cu1-N1	-153.7(2)
C5-C4-N1-C1	-172.0(3)	C9-N2-Cu1-N3	26.9(2)
C5-C4-N1-Cu1	38.1(3)	C9-N2-Cu1-Cu2	88.5(4)
C5-C6-C7-C8	-174.2(3)	C9-N2-Cu1-O1	-50.0(3)
C5-C6-C7-C39	3.1(6)	C9-N2-Cu1-O3	116.3(2)
C5-C6-N2-C9	174.1(3)	C9-N2-Cu1-Cl3	-46.0(4)
C5-C6-N2-Cu1	20.3(4)	C10-C9-N2-C6	-177.8(3)
C5-C33-C34-C35	179.7(3)	C10-C9-N2-Cu1	-18.6(3)
C5-C33-C38-C37	-178.2(3)	C10-C11-C12-C13	-4.9(5)
C6-C5-C33-C34	112.8(3)	C11-C10-N3-C14	6.4(4)
C6-C5-C33-C38	-67.7(4)	C11-C10-N3-Cu1	-149.8(2)
C6-C7-C8-C9	0.0(4)	C11-C12-C13-C14	4.1(5)
C6-C7-C8-C41	176.5(3)	C12-C13-C14-C15	-173.1(3)

C12-C13-C14-N3	2.0(5)	C19-C47-C52-C51	177.7(3)
C13-C14-C15-C16	53.2(4)	C20-C19-C47-C48	99.8(3)
C13-C14-C15-N4	-116.0(3)	C20-C19-C47-C52	-77.5(4)
C13-C14-N3-C10	-7.1(4)	C20-C21-C22-C23	-1.5(3)
C13-C14-N3-Cu1	142.7(2)	C20-C21-C22-C55	177.7(3)
C14-C15-C16-C17	-169.3(3)	C20-C21-C53-C54	100.6(4)
C14-C15-C16-C43	15.6(5)	C20-N5-Cu2-N4	28.7(3)
C14-C15-N4-C18	166.6(3)	C20-N5-Cu2-N6	-162.0(3)
C14-C15-N4-Cu2	-46.7(4)	C20-N5-Cu2-Cu1	140.7(2)
C15-C14-N3-C10	167.5(3)	C20-N5-Cu2-O1	126.6(3)
C15-C14-N3-Cu1	-42.6(4)	C20-N5-Cu2-O3	-67.9(3)
C15-C16-C17-C18	0.9(3)	C20-N5-Cu2-Cl3	120.3(4)
C15-C16-C17-C45	-173.6(3)	C21-C20-N5-C23	0.8(3)
C15-C16-C43-C44	63.9(4)	C21-C20-N5-Cu2	170.4(2)
C16-C15-N4-C18	-3.3(3)	C21-C22-C23-C24	-171.0(3)
C16-C15-N4-Cu2	143.3(2)	C21-C22-C23-N5	2.0(3)
C16-C17-C18-C19	172.1(3)	C21-C22-C55-C56	-93.9(4)
C16-C17-C18-N4	-2.9(3)	C22-C21-C53-C54	-79.1(4)
C16-C17-C45-C46	87.6(4)	C22-C23-C24-C25	-23.0(5)
C17-C16-C43-C44	-110.2(4)	C22-C23-C24-N6	157.7(3)
C17-C18-C19-C20	178.2(3)	C22-C23-N5-C20	-1.8(3)
C17-C18-C19-C47	-4.3(5)	C22-C23-N5-Cu2	-172.71(19)
C17-C18-N4-C15	3.8(3)	C23-C22-C55-C56	85.2(4)
C17-C18-N4-Cu2	-146.9(2)	C23-C24-C25-C26	-177.6(3)
C18-C17-C45-C46	-85.5(4)	C23-C24-N6-C28	-179.7(2)
C18-C19-C20-C21	171.8(3)	C23-C24-N6-Cu2	19.1(3)
C18-C19-C20-N5	-10.5(4)	C23-N5-Cu2-N4	-162.5(2)
C18-C19-C47-C48	-77.9(4)	C23-N5-Cu2-N6	6.8(2)
C18-C19-C47-C52	104.8(3)	C23-N5-Cu2-Cu1	-50.5(4)
C19-C18-N4-C15	-171.4(3)	C23-N5-Cu2-O1	-64.6(3)
C19-C18-N4-Cu2	37.8(3)	C23-N5-Cu2-O3	100.9(3)
C19-C20-C21-C22	178.4(3)	C23-N5-Cu2-Cl3	-70.9(4)
C19-C20-C21-C53	-1.4(6)	C24-C23-N5-C20	172.9(2)
C19-C20-N5-C23	-177.4(3)	C24-C23-N5-Cu2	1.9(3)
C19-C20-N5-Cu2	-7.8(4)	C24-C25-C26-C27	-1.9(5)
C19-C47-C48-C49	-177.1(3)	C25-C24-N6-C28	1.0(4)

C25-C24-N6-Cu2	-160.3(2)	C50-C51-C52-C47	-0.7(5)
C25-C26-C27-C28	-0.4(5)	C52-C47-C48-C49	0.2(5)
C26-C27-C28-C1	-169.0(3)	C53-C21-C22-C23	178.3(3)
C26-C27-C28-N6	3.1(5)	C53-C21-C22-C55	-2.5(5)
C27-C28-N6-C24	-3.3(4)	C55-C22-C23-C24	9.9(6)
C27-C28-N6-Cu2	153.8(2)	C55-C22-C23-N5	-177.1(3)
C28-C1-C2-C3	-169.7(2)	N1-C1-C2-C3	1.7(3)
C28-C1-C2-C29	12.2(4)	N1-C1-C2-C29	-176.4(3)
C28-C1-N1-C4	167.3(3)	N1-C1-C28-C27	-112.8(3)
C28-C1-N1-Cu1	-44.7(4)	N1-C1-C28-N6	75.3(4)
C29-C2-C3-C4	178.7(3)	N1-C4-C5-C6	-15.6(4)
C29-C2-C3-C31	7.5(5)	N1-C4-C5-C33	161.4(3)
C31-C3-C4-C5	-16.7(5)	N2-C6-C7-C8	1.1(3)
C31-C3-C4-N1	167.9(3)	N2-C6-C7-C39	178.4(3)
C33-C5-C6-C7	-16.8(5)	N2-C9-C10-C11	168.9(3)
C33-C5-C6-N2	168.3(3)	N2-C9-C10-N3	-8.6(4)
C33-C34-C35-C36	-1.3(5)	N3-C10-C11-C12	-0.3(5)
C34-C33-C38-C37	1.3(5)	N3-C14-C15-C16	-121.7(3)
C34-C35-C36-C37	0.9(5)	N3-C14-C15-N4	69.2(4)
C35-C36-C37-C38	0.6(6)	N4-C15-C16-C17	1.6(4)
C36-C37-C38-C33	-1.7(6)	N4-C15-C16-C43	-173.6(3)
C38-C33-C34-C35	0.2(5)	N4-C18-C19-C20	-7.6(5)
C39-C7-C8-C9	-177.4(3)	N4-C18-C19-C47	169.9(3)
C39-C7-C8-C41	-0.9(5)	N5-C20-C21-C22	0.5(3)
C41-C8-C9-C10	2.1(6)	N5-C20-C21-C53	-179.2(3)
C41-C8-C9-N2	-177.5(3)	N5-C23-C24-C25	164.3(3)
C43-C16-C17-C18	175.9(3)	N5-C23-C24-N6	-15.0(4)
C43-C16-C17-C45	1.4(5)	N6-C24-C25-C26	1.7(4)
C45-C17-C18-C19	-14.0(5)	Cu1-O1-C57-O2	5.6(4)
C45-C17-C18-N4	171.1(3)	Cu1-O1-C57-C58	-173.7(3)
C47-C19-C20-C21	-5.8(5)	Cu2-O1-C57-O2	-143.4(3)
C47-C19-C20-N5	171.9(3)	Cu2-O1-C57-C58	37.2(4)
C47-C48-C49-C50	-0.4(5)	O4-C59-O3-Cu1	134.4(9)
C48-C47-C52-C51	0.4(5)	O4-C59-O3-Cu2	-3.9(9)
C48-C49-C50-C51	0.0(5)	C60-C59-O3-Cu1	-45.6(8)
C49-C50-C51-C52	0.5(5)	C60-C59-O3-Cu2	176.0(8)

O4A-C59-O3-Cu1	150.0(5)	C60A-C59-O3-Cu1	-29.6(5)
O4A-C59-O3-Cu2	11.6(4)	C60A-C59-O3-Cu2	-167.9

Supporting References

(1) CrysAlis Pro. Rigaku Oxford Diffraction, CrysAlisPro Software System, 1.171.40.37a, 2019.

(2) Sheldrick, G. M. SHELXT. A Program for crystal structure solution. *Acta Cryst.* **2015**, *A71*, 3-8.

(3) Sheldrick, G. M. SHELXL-2016/6. Program for the Refinement of Crystal Structures. *Acta Cryst.* **2015**, *C71*, 9-18.

(4) Spek, A. L. PLANTON, A Multipurpose Crystallographic Tool. *Acta Cryst.* **2009**, *D65*, 138-143.

(5) Dolomanov, O. V.; Bourhis, L. J.; Gildea, R. J.; Howard, J. A. K.; Puschmann, H. A Complete Structure Solution, Refinement, and Analysis Program. OLEX2. *J Appl. Cryst.* **2009**, *42*, 339-341.

(6) Farrugia, L. J. J. WinGX 1.64. An Integrated System of Windows Programs for the Solution, Refinement and Analysis of Single Crystal X-ray Diffraction Data. *Appl. Cryst.* **1999**, *32*, 837-838.

(7) $R_W(F^2) = \{\Sigma w(|F_o|^2 - |F_c|^2)^2/\Sigma w(|F_o|)^4\}^{1/2}$ where w is the weight given each reflection. $R(F) = \Sigma(|F_o| - |F_c|)/\Sigma|F_o|\}$ for reflections with $F_o > 4(\sigma(F_o))$. $S = [\Sigma w(|F_o|^2 - |F_c|^2)^2/(n - p)]^{1/2}$, where n is the number of reflections and p is the number of refined parameters.

(8) Wilson, A. J. C., International Tables for X-ray Crystallography. Vol. C, Tables 4.2.6.8 and 6.1.1.4. Boston: Kluwer Academic Press, 1992.

(9) Sheldrich, G. M. SHELXTL/PC (Version 5.03). Siemens Analytical X-ray Instruments, Inc. Madison, Wisconsin USA, 1994.

checkCIF/PLATON report

Structure factors have been supplied for datablock(s) jtb-dpam-biscu-x2-redo

THIS REPORT IS FOR GUIDANCE ONLY. IF USED AS PART OF A REVIEW PROCEDURE FOR PUBLICATION, IT SHOULD NOT REPLACE THE EXPERTISE OF AN EXPERIENCED CRYSTALLOGRAPHIC REFEREE.

No syntax errors found. CIF dictionary Interpreting this report

Datablock: jtb-dpam-biscu-x2-redo

```
Bond precision:  C-C = 0.0049 A          Wavelength=1.54184

Cell:          a=12.7793(4)       b=14.4880(6)     c=17.7112(5)
               alpha=112.327(3)   beta=95.763(3)   gamma=110.276(3)
Temperature:   100 K
```

	Calculated	Reported
Volume	2741.7(2)	2741.73(17)
Space group	P -1	P -1
Hall group	-P 1	-P 1
Moiety formula	2(C59.78 H61.67 Cl0.11 Cu2 N6 O3.78), 2(C H2 Cl2), H2 O	C59.78 H61.67 Cl0.11 Cu2 N6 O3.78, C H2 Cl2, 0.5(H2 O)
Sum formula	C121.56 H129.34 Cl4.22 Cu4 N12 O8.56	C61 H65 Cl2 Cu2 N6 O4.50
Mr	2299.19	1152.17
Dx,g cm-3	1.393	1.396
Z	1	2
Mu (mm-1)	2.331	2.287
F000	1198.9	1202.0
F000'	1195.38	
h,k,lmax	15,17,21	15,17,21
Nref	10047	10031
Tmin,Tmax	0.801,0.947	0.631,1.000
Tmin'	0.605	

```
Correction method= # Reported T Limits: Tmin=0.631 Tmax=1.000
AbsCorr = 'G

Data completeness= 0.998          Theta(max)= 68.244

R(reflections)= 0.0551( 8252)     wR2(reflections)= 0.1524( 10031)

S = 1.022                   Npar= 742
```

The following ALERTS were generated. Each ALERT has the format
test-name_ALERT_alert-type_alert-level.
Click on the hyperlinks for more details of the test.

Alert level C

```
ABSTY02_ALERT_1_C  An _exptl_absorpt_correction_type has been given without
            a literature citation. This should be contained in the
            _exptl_absorpt_process_details field.
            Absorption correction given as gaussian and multi-scan
PLAT041_ALERT_1_C Calc. and Reported SumFormula    Strings  Differ     Please Check
PLAT043_ALERT_1_C Calculated and Reported Mol. Weight Differ by ..       5.15 Check
PLAT051_ALERT_1_C Mu(calc) and Mu(CIF) Ratio Differs from 1.0 by .       1.92 %
PLAT068_ALERT_1_C Reported F000 Differs from Calcd (or Missing)...     Please Check
PLAT077_ALERT_4_C Unitcell Contains Non-integer Number of Atoms ..     Please Check
PLAT213_ALERT_2_C Atom Cl3             has ADP max/min Ratio .....        3.8 oblate
PLAT336_ALERT_2_C Long Bond Distance for ..... C1B      -Cl2A           1.890 Ang.
PLAT906_ALERT_3_C Large K Value in the Analysis of Variance ......      2.143 Check
PLAT911_ALERT_3_C Missing FCF Refl Between Thmin & STh/L=    0.600         16 Report
```

Alert level G

```
FORMU01_ALERT_1_G  There is a discrepancy between the atom counts in the
            _chemical_formula_sum and _chemical_formula_moiety. This is
            usually due to the moiety formula being in the wrong format.
            Atom count from _chemical_formula_sum:   C61 H65 Cl2 Cu2 N6 O4.5
            Atom count from _chemical_formula_moiety:C60.78 H64.67 Cl2.11 Cu2 N6 O
FORMU01_ALERT_2_G  There is a discrepancy between the atom counts in the
            _chemical_formula_sum and the formula from the _atom_site* data.
            Atom count from _chemical_formula_sum:C61 H65 Cl2 Cu2 N6 O4.5
            Atom count from the _atom_site data:  C60.78 H64.67 Cl2.11 Cu2 N6 O4.2
ABSTY01_ALERT_1_G  Extra text has been found in the _exptl_absorpt_correction_type
            field, which should be only a single keyword. A literature
            citation should be included in the _exptl_absorpt_process_details
            field.
CELLZ01_ALERT_1_G Difference between formula and atom_site contents detected.
CELLZ01_ALERT_1_G ALERT: Large difference may be due to a
            symmetry error - see SYMMG tests
           From the CIF: _cell_formula_units_Z    2
           From the CIF: _chemical_formula_sum  C61 H65 Cl2 Cu2 N6 O4.50
           TEST: Compare cell contents of formula and atom_site data

           atom    Z*formula  cif sites diff
           C        122.00    121.56    0.44
           H        130.00    129.34    0.66
           Cl         4.00      4.22   -0.22
           Cu         4.00      4.00    0.00
           N         12.00     12.00    0.00
           O          9.00      8.56    0.44
PLAT002_ALERT_2_G Number of Distance or Angle Restraints on AtSite         14 Note
PLAT003_ALERT_2_G Number of Uiso or Uij Restrained non-H Atoms ...         12 Report
PLAT007_ALERT_5_G Number of Unrefined Donor-H Atoms .............          2 Report
PLAT042_ALERT_1_G Calc. and Reported MoietyFormula Strings  Differ     Please Check
PLAT045_ALERT_1_G Calculated and Reported Z Differ by a Factor ...       0.50 Check
PLAT152_ALERT_1_G The Supplied and Calc. Volume s.u. Differ by ...          3 Units
PLAT154_ALERT_1_G The s.u.'s on the Cell Angles are Equal ..(Note)      0.003 Degree
PLAT171_ALERT_4_G The CIF-Embedded .res File Contains EADP Records          2 Report
PLAT172_ALERT_4_G The CIF-Embedded .res File Contains DFIX Records          2 Report
PLAT174_ALERT_4_G The CIF-Embedded .res File Contains FLAT Records          3 Report
PLAT176_ALERT_4_G The CIF-Embedded .res File Contains SADI Records          3 Report
```

```
PLAT178_ALERT_4_G The CIF-Embedded .res File Contains SIMU Records          3 Report
PLAT186_ALERT_4_G The CIF-Embedded .res File Contains ISOR Records          1 Report
PLAT230_ALERT_2_G Hirshfeld Test Diff for    O2       --C57      .        5.5 s.u.
PLAT232_ALERT_2_G Hirshfeld Test Diff (M-X)  Cu1      --O1       .        7.8 s.u.
PLAT232_ALERT_2_G Hirshfeld Test Diff (M-X)  Cu1      --O3       .        5.6 s.u.
PLAT232_ALERT_2_G Hirshfeld Test Diff (M-X)  Cu2      --O1       .        5.5 s.u.
PLAT300_ALERT_4_G Atom Site Occupancy of O4A        Constrained at       0.68 Check
PLAT300_ALERT_4_G Atom Site Occupancy of O4         Constrained at       0.32 Check
PLAT300_ALERT_4_G Atom Site Occupancy of C60A       Constrained at       0.68 Check
PLAT300_ALERT_4_G Atom Site Occupancy of C60        Constrained at       0.32 Check
PLAT300_ALERT_4_G Atom Site Occupancy of H60D       Constrained at       0.68 Check
PLAT300_ALERT_4_G Atom Site Occupancy of H60E       Constrained at       0.68 Check
PLAT300_ALERT_4_G Atom Site Occupancy of H60F       Constrained at       0.68 Check
PLAT300_ALERT_4_G Atom Site Occupancy of H60A       Constrained at       0.32 Check
PLAT300_ALERT_4_G Atom Site Occupancy of H60B       Constrained at       0.32 Check
PLAT300_ALERT_4_G Atom Site Occupancy of H60C       Constrained at       0.32 Check
PLAT300_ALERT_4_G Atom Site Occupancy of Cl1        Constrained at       0.52 Check
PLAT300_ALERT_4_G Atom Site Occupancy of Cl2        Constrained at       0.52 Check
PLAT300_ALERT_4_G Atom Site Occupancy of C1A        Constrained at       0.52 Check
PLAT300_ALERT_4_G Atom Site Occupancy of H1AA       Constrained at       0.52 Check
PLAT300_ALERT_4_G Atom Site Occupancy of H1AB       Constrained at       0.52 Check
PLAT300_ALERT_4_G Atom Site Occupancy of Cl1A       Constrained at       0.48 Check
PLAT300_ALERT_4_G Atom Site Occupancy of Cl2A       Constrained at       0.48 Check
PLAT300_ALERT_4_G Atom Site Occupancy of C1B        Constrained at       0.48 Check
PLAT300_ALERT_4_G Atom Site Occupancy of H1BA       Constrained at       0.48 Check
PLAT300_ALERT_4_G Atom Site Occupancy of H1BB       Constrained at       0.48 Check
PLAT300_ALERT_4_G Atom Site Occupancy of O1W        Constrained at        0.5 Check
PLAT300_ALERT_4_G Atom Site Occupancy of H1WA       Constrained at        0.5 Check
PLAT300_ALERT_4_G Atom Site Occupancy of H1WB       Constrained at        0.5 Check
PLAT301_ALERT_3_G Main Residue  Disorder .............(Resd  1  )         8% Note
PLAT302_ALERT_4_G Anion/Solvent/Minor-Residue Disorder (Resd  2  )       100% Note
PLAT302_ALERT_4_G Anion/Solvent/Minor-Residue Disorder (Resd  3  )       100% Note
PLAT302_ALERT_4_G Anion/Solvent/Minor-Residue Disorder (Resd  4  )       100% Note
PLAT304_ALERT_4_G Non-Integer Number of Atoms in ..... (Resd  1  )     133.34 Check
PLAT304_ALERT_4_G Non-Integer Number of Atoms in ..... (Resd  2  )       2.60 Check
PLAT304_ALERT_4_G Non-Integer Number of Atoms in ..... (Resd  3  )       2.40 Check
PLAT304_ALERT_4_G Non-Integer Number of Atoms in ..... (Resd  4  )       1.50 Check
PLAT432_ALERT_2_G Short Inter X...Y Contact  O1W      ..C1A              3.00 Ang.
                                               1-x,1-y,1-z  =      2_666 Check
PLAT720_ALERT_4_G Number of Unusual/Non-Standard Labels ..........          6 Note
PLAT802_ALERT_4_G CIF Input Record(s) with more than 80 Characters         14 Info
PLAT860_ALERT_3_G Number of Least-Squares Restraints .............        120 Note
PLAT883_ALERT_1_G No Info/Value for _atom_sites_solution_primary .     Please Do !
PLAT978_ALERT_2_G Number C-C Bonds with Positive Residual Density.          3 Info
```

---

   0 **ALERT level A** = Most likely a serious problem - resolve or explain
   0 **ALERT level B** = A potentially serious problem, consider carefully
  10 **ALERT level C** = Check. Ensure it is not caused by an omission or oversight
  59 **ALERT level G** = General information/check it is not something unexpected

  14 ALERT type 1 CIF construction/syntax error, inconsistent or missing data
  11 ALERT type 2 Indicator that the structure model may be wrong or deficient
   4 ALERT type 3 Indicator that the structure quality may be low
  39 ALERT type 4 Improvement, methodology, query or suggestion
   1 ALERT type 5 Informative message, check

It is advisable to attempt to resolve as many as possible of the alerts in all categories. Often the minor alerts point to easily fixed oversights, errors and omissions in your CIF or refinement strategy, so attention to these fine details can be worthwhile. In order to resolve some of the more serious problems it may be necessary to carry out additional measurements or structure refinements. However, the purpose of your study may justify the reported deviations and the more serious of these should normally be commented upon in the discussion or experimental section of a paper or in the "special_details" fields of the CIF. checkCIF was carefully designed to identify outliers and unusual parameters, but every test has its limitations and alerts that are not important in a particular case may appear. Conversely, the absence of alerts does not guarantee there are no aspects of the results needing attention. It is up to the individual to critically assess their own results and, if necessary, seek expert advice.

Publication of your CIF in IUCr journals

A basic structural check has been run on your CIF. These basic checks will be run on all CIFs submitted for publication in IUCr journals (*Acta Crystallographica*, *Journal of Applied Crystallography*, *Journal of Synchrotron Radiation*); however, if you intend to submit to *Acta Crystallographica Section C* or *E* or *IUCrData*, you should make sure that full publication checks are run on the final version of your CIF prior to submission.

Publication of your CIF in other journals

Please refer to the *Notes for Authors* of the relevant journal for any special instructions relating to CIF submission.

PLATON version of 22/12/2019; check.def file version of 13/12/2019

Datablock jtb-dpam-biscu-x2-redo - ellipsoid plot

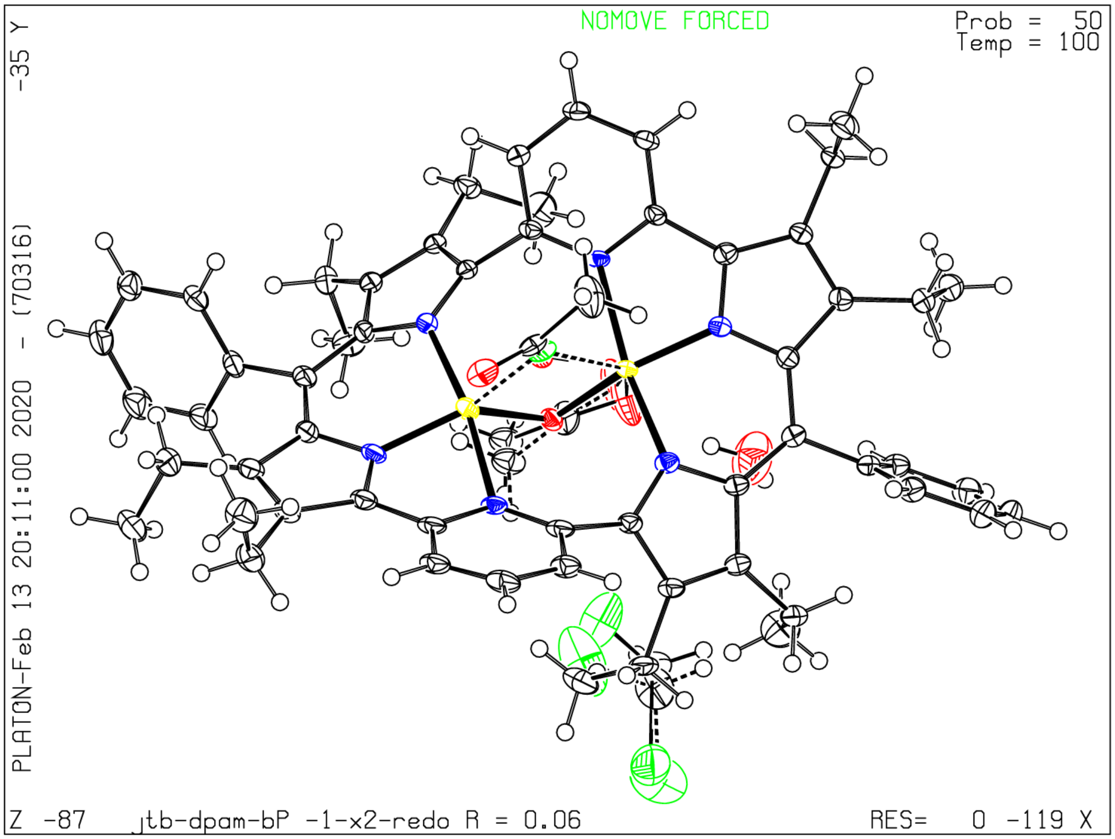

Supplement: Supplementary file 1 [file molecules-25-01446-s001.pdf]
